# The Use of Biopolymers as a Natural Matrix for Incorporation of Essential Oils of Medicinal Plants

**DOI:** 10.3390/gels8110756

**Published:** 2022-11-21

**Authors:** Roxana Gheorghita Puscaselu, Andrei Lobiuc, Ioan Ovidiu Sirbu, Mihai Covasa

**Affiliations:** 1 Department of Biochemistry, Victor Babeş University of Medicine and Pharmacy, 300041 Timisoara, Romania; 2 Department of Medicine and Biomedical Sciences, College of Medicine and Biological Science, University of Suceava, 720229 Suceava, Romania; 3 Center for Complex Network Science, Victor Babes University of Medicine and Pharmacy, 300041 Timisoara, Romania; 4 Department of Basic Medical Sciences, College of Osteopathic Medicine, Western University of Health Sciences, Pomona, CA 91766, USA

**Keywords:** biopolymers, citrus, cinnamon, clove, eucalyptus, mint, ginger, chamomile

## Abstract

The benefits of using biopolymers for the development of films and coatings are well known. The enrichment of these material properties through various natural additions has led to their applicability in various fields. Essential oils, which are well-known for their beneficial properties, are widely used as encapsulating agents in films based on biopolymers. In this study, we developed biopolymer-based films and tested their properties following the addition of 7.5% and 15% (*w*/*v*) essential oils of lemon, orange, grapefruit, cinnamon, clove, chamomile, ginger, eucalyptus or mint. The samples were tested immediately after development and after one year of storage in order to examine possible long-term property changes. All films showed reductions in mass, thickness and microstructure, as well as mechanical properties. The most considerable variations in physical properties were observed in the 7.5% lemon oil sample and the 15% grapefruit oil sample, with the largest reductions in mass (23.13%), thickness (from 109.67 µm to 81.67 µm) and density (from 0.75 g/cm^3^ to 0.43 g/cm^3^). However, the microstructure of the sample was considerably improved. Although the addition of lemon essential oil prevented the reduction in mass during the storage period, it favored the degradation of the microstructure and the loss of elasticity (from 16.7% to 1.51% for the sample with 7.5% lemon EO and from 18.28% to 1.91% for the sample with 15% lemon EO). Although the addition of essential oils of mint and ginger resulted in films with a more homogeneous microstructure, the increase in concentration favored the appearance of pores and modifications of color parameters. With the exception of films with added orange, cinnamon and clove EOs, the antioxidant capacity of the films decreased during storage. The most obvious variations were identified in the samples with lemon, mint and clove EOs. The most unstable samples were those with added ginger (95.01%), lemon (92%) and mint (90.22%).

## 1. Introduction

For the past decade, the use of biopolymers materials instead of conventional and highly polluting materials, has increased substantially. Biopolymers can be obtained from various sources; the most used are those based on polysaccharides (sodium alginate, agar, chitosan, carrageenan, starch and cellulose), lipids (waxes and fatty acids) and proteins (gelatin, collagen and soy protein isolate) [1]. They are extensively used, owing to the properties of biobased materials, such as mechanical performance, physicochemical characteristics comparable to those of conventional materials [2], antibacterial and antioxidant properties, high biocompatibility, compostability, non-toxicity, non-immunogenicity, non-carcinogenicity, and non-inflammatory and non-allergenic character [3,4]. Multiple manufacturing methods have increased their widespread use. They can be used for the development of films or coatings, capsules, gels or hydrogels. Owing to their matrix, which has a compact structure and high retention capacity, they can be enriched by the addition of various biologically active substances. Furthermore, they are environmentally friendly, completely edible and produce zero waste after use [5].

Essential oils (EOs) have been used since ancient times, owing to their health benefits and sensory and antioxidant properties. Thus, essential oils, such as those extracted from medicinal plants, contain active compounds, such as limonene, pinene, myrcene (orange, lemon, grapefruit and chamomile) [6,7], eucalyptol, eugenol, gingerol, and cedrene (ginger, cinnamon, clove and mint) compound [8], with beneficial effects on the human body. Thus, orange, lemon and ginger EOs have been used for their relaxant effect or pain relief [9,10], whereas grapefruit, cinnamon and mint EOs have been used for their anti-inflammatory, antimycotoxigenic, antitumoral and antigenotoxic effects [11,12,13]. In medicine, lemon EO has been used to relieve the symptoms of gastrointestinal diseases and depression [14]; ginger EO has been used for sore throat, cough, cold, dyspepsia, gastritis and gastric ulcerations [15]; clove EO has been used for urinary tract infections, digestive disorders, athlete’s foot disease, in dentistry and as a kidney tonic [16,17].

Several studies have demonstrated the positive effects of the use of essential oils, for example, in preventing infection with the SARS-CoV-2 virus. Senthil Kumar et al. [18] showed that lemon essential oil, through its compounds, such as citronellol, geraniol, and neryl acetate, was capable of preventing replication of coronaviruses by blocking the entry of the virus into the host cells through downregulation of ACE2 receptor expression in epithelial cells.

Owing to their benefits and consumers predisposition toward products that are as natural as possible, research in the field has been oriented towards the development of innovative materials obtained from renewable resources. Thus, materials based on biopolymers with incorporated essential oils have been developed. Initially, they were used in the food industry to improve the sensorial quality or to increase the shelf life of various food products, such as those based on meat or fish [19,20,21,22], fruits or vegetables [23,24,25], sweets [26,27], beverages [28] or dairy products [29,30]. Later, they were added to increase the nutritional value and bioavailability of products, especially those with beneficial effects on health. Thus, numerous essential oils, such as those from citrus fruits (lemon, orange and grapefruit) or medicinal EOs (ginger, chamomile, mint, eucalyptus, cinnamon or cloves) have been incorporated into biopolymeric materials. However, the development, characterization and testing of these films under various experimental conditions using several well-known plant-based oils with applicability in biomedical, cosmetic or food industries are still needed. Therefore, in this study, we tested biofilms incorporated with two concentrations (7.5% and 15%, *v*/*w*) of essential oils of lemon, orange, grapefruit, cinnamon, clove, chamomile, ginger, eucalyptus or mint at the time of development and after one year of storage under normal temperature and humidity conditions.

## 2. Results and Discussion

**Figure 1:**
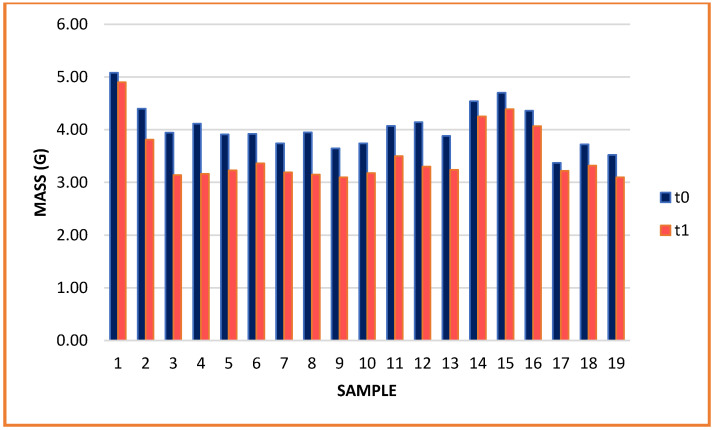
Mass of biopolymeric films.

In the present study, we examined the preservation capacity of biopolymeric films with various incorporated plant-derived essential oils. Numerous studies have examined the benefits of using films and coatings based on biopolymers and essential oils; however, the long-term effect on their properties is not entirely known. Our results show that the storage of films considerably influences their properties. Although the films were kept under temperature- and humidity-controlled conditions, their mass varied after one year (Figure 1).

**Figure 2:**
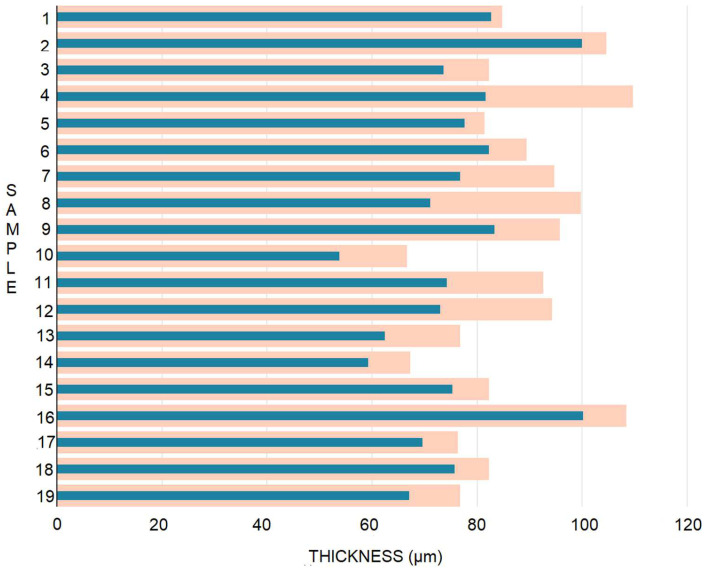
Sample thickness after one year of storage. T0, light brown bars; T1, blue bars.

As depicted in Figure 1, the mass of all samples was reduced during the test period. Although samples were kept in silicone paper packaging, simulating a real storage environment, significant moisture loss had occurred after one year. The highest mass reduction was observed in sample 4 with 15% grapefruit EO (23.13%), and the lowest mass reduction was observed in sample 1 with 15% lemon EO (3.54%). The mass loss was associated with a significant reduction in film thickness (Figure 2).

The thickness of the films did not undergo major changes after one year compared to the initial period. The largest variation was observed in the films with the addition of cinnamon (from 94.67 to 76.67 µm and 99.67 to 71.00 µm, respectively) and cloves (from 95.67 µm to 83.33 µm (9) and from 66.67 µm to 53.67 µm for sample 10) oils. The thickness of the control sample was reduced by 9.7 µm. The determination and the results are of interest to developers who may want to produce such materials on a large scale.

The mass reduction in the films can be attributed to the elimination of water from the film matrix, which was also indicated by the reduced values of the water activity index. These values decreased by at least 50% for all samples (Table 1). A decrease in the water activity index can be beneficial in terms of preventing the development of microorganisms (which require at least a_w_ > 0.7), but it can also alter the film microstructure, making it more brittle and fragile. 

**Figure 3:**
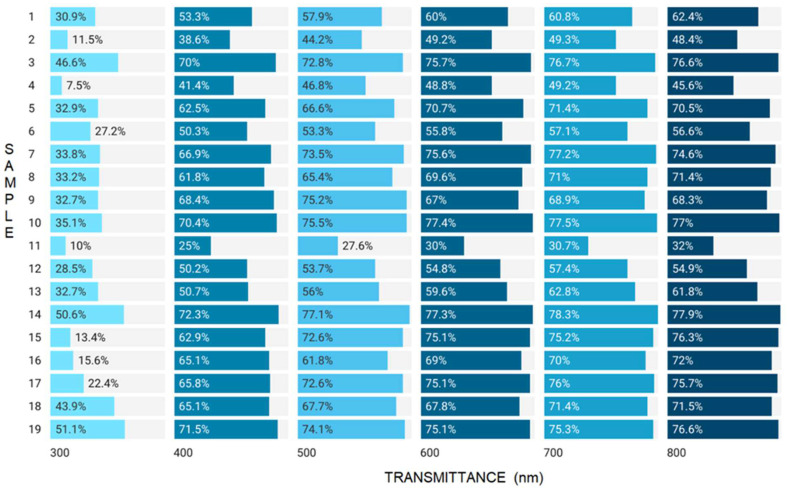
Transmittance values of samples (t0).

**Figure 4:**
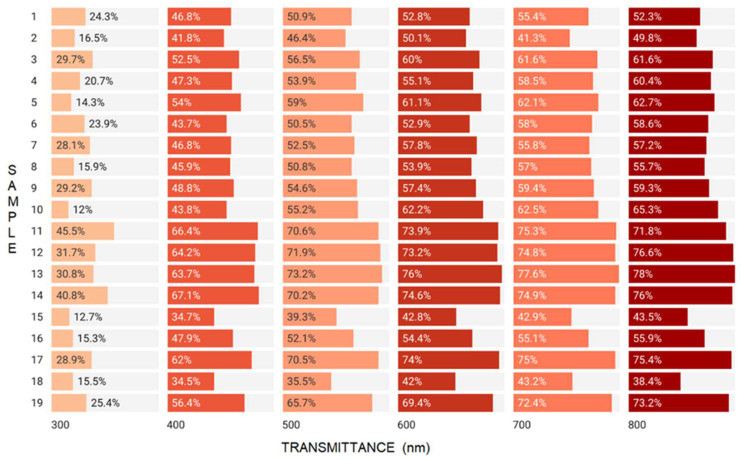
The transmittance values of samples after one year of storage (t1).

In addition to dehydration, a reduction in sample density also occurred, totaling approximately one unit for samples 5, 6 and 14–18 with incorporated orange, chamomile, ginger and eucalyptus essential oils. Additionally, the density of the control sample (19) decreased by one unit (from 0.44 to 0.34 g/cm^3^). The greatest reduction in density was observed in sample 4 (56.75%) composed of 15% grapefruit essential oil. Except for grapefruit (3, 4), mint (11, 12) and the control (19), all films exhibited increased opacity after one year of storage. These results were confirmed by a corresponding decrease in the transmittance parameter evaluated before and after storage (Figure 3 and Figure 4).

As depicted in Figure 3, the transmittance values increased within 300–700 nm range. At 800 nm (visible light), films with 7.5% lemon (1), 15% cinnamon (8), 7.5% mint (11), 7.5 and 15% ginger (15,16), 15% eucalyptus (18) EOs and the control (19) exhibited increased transmittance, whereas in the remainder of the films, the transmission was decreased. This determination is important because it evaluates the ability of the material to resist UV radiation can degrade the incorporated product and even the foil, especially when it contains essential oil. The smallest variations were observed in the control sample (19) both before and after storage. After storage, the films were more resistant to the action of UV radiation, an effect strengthened by the increased opacity values after storage (Table 1). 

The sample color was also influenced by storage. Except for samples with added mint oil (11, 12), in which the luminosity values increased during the storage period (from 90.61 and 91.9 to 92.25 and 92.18, respectively), for all other samples, the values were reduced but did not change significantly. The color deviation was more evident for samples 1–10 (ΔE = 6.24–7.55) (Table 2). For samples 11–17, ΔE did not exceed 3.63. The smallest color deviation was observed in sample 12 with 15% mint oil (ΔE = 0.244), and the largest color deviation was observed in sample 18 with 15% eucalyptus oil (ΔE = 11.35). 

With some exceptions, the surface morphology of films with added oils was influenced by prolonged storage (Figure S1, Supplementary Files). The microstructure of films with 7.5 and 15% cinnamon (7,8), 7.5 and 15% clove (9,10) and 15% chamomile essential oils was negatively modified during storage. Changes also occurred in the structure of the control sample. The film with 15% grapefruit EO became rougher, indicating changes in the matrix, which was more homogeneous and compact prior to storage. The same pattern was observed for samples with 15% concentration of orange (6) and cinnamon (8) EOs. The film with 15% added clove oil (10) exhibited pores in its microstructure.

Except for samples with added mint and ginger oil (11–14), all other films with essential oil presented a less homogeneous microstructure than the control sample. Thus, in order to obtain materials with superior physical properties, the matrix could be improved by increasing the amount of emulsifier in the film-forming solution (i.e., Tween 80).

**Figure 5:**
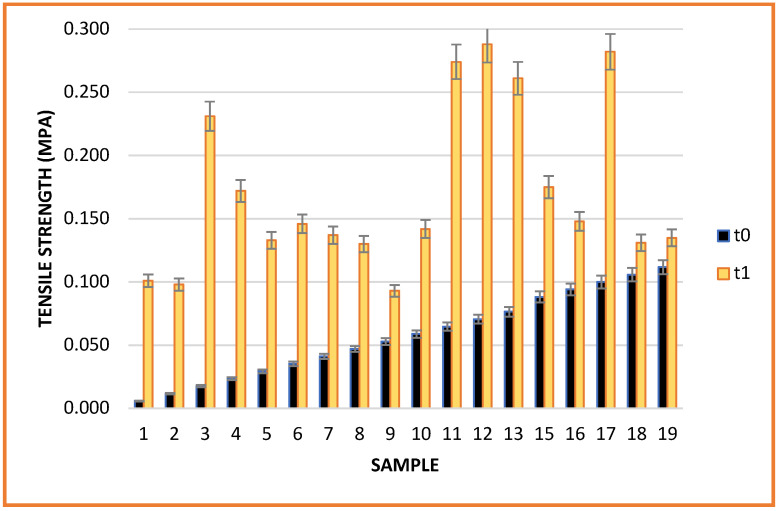
Sample tensile strength.

The mechanical characteristics of the films were not influenced by the storage period. All samples subjected to testing showed a increased tensile strength after storage (Figure 5). The increase in breaking strength may be due to the amount of essential oil lost during storage in association with the overall decrease in the antioxidant activity of most films with added oil. The breaking strength of biopolymeric films has been shown to be affected by the addition of natural substances. For example, the mechanical performance was decreased with increasing content of natural substances added to films [31,32]. This effect was attributed to the plasticizing effect of EOs weakening the intermolecular interaction between polymer chains and increasing the ductility of the film [32,33].

Sample 14 (15% ginger) could not be tested to evaluate its mechanical properties as a result of the drying method used. Thus, a 15% concentration of ginger oil may require a increased amount of plasticizer or emulsifier in the composition.

**Figure 6:**
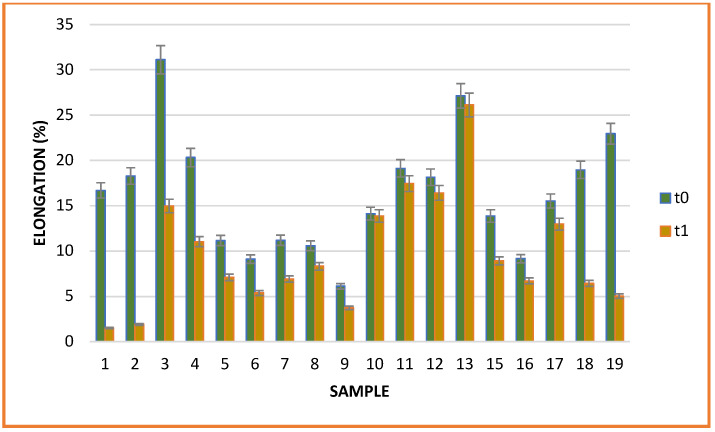
Sample elongation.

As expected, the elongation at break of the films was negatively influenced during the storage period. The loss of water from the films unbalanced the matrix, so despite strengthened tear resistance, they showed considerably reduced elasticity (Figure 6).

The largest variation in elongation was observed in the lemon oil samples (16.7 (t0) to 1.51% (t1) and 18.28 (t0) to 1.91% (t1) for samples 1 and 2, respectively), regardless of the concentration. Large variations were also observed in the grapefruit, ginger and eucalyptus samples, with variations ranging between 37% and 50%. High variation was also observed in the control sample without the addition of essential oil. Therefore, the dehydration of the material did not depend on the nature of oil added but on the biopolymeric composition or environmental factors. However, the addition of orange, cinnamon, clove, mint and chamomile essential oils maintained the elasticity of the samples, with minimal change relative to the initial period.

**Figure 7:**
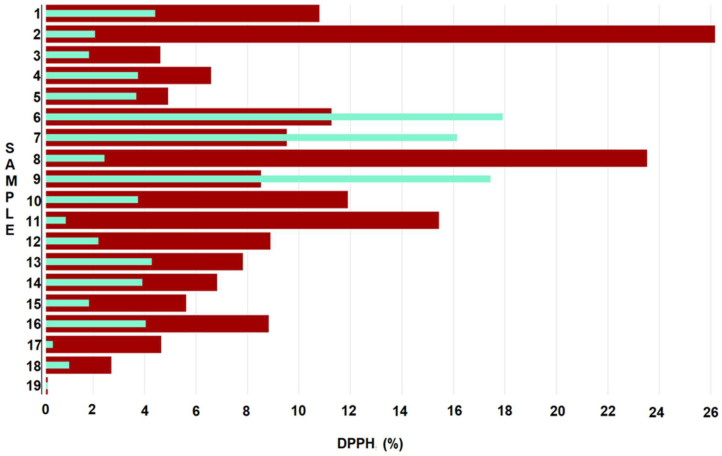
Graphical representation of DPPH radical scavenging activity of biopolymeric films before (red) and after storage (turquoise).

The antioxidant activity of most samples was significantly diminished after one year of storage (Figure 7). For example, the antioxidant activity of sample 2 with 15% lemon oil decreased from 26% to 1.9%, which was the most significant decrease among all samples tested. A significant reduction in the antioxidant capacity was also observed in sample 8 with 15% cinnamon oil (from 23.36% to 2.28%). A modest decrease was also observed in sample 5 (7.5% orange oil) when the antioxidant activity dropped from 4.72% at *t*0 to 3.5% at *t*1. However, for a few samples, the antioxidant activity of the films increased with the addition of 15% orange (6), 7.5% cinnamon (7) and 7.5% clove (9) essential oils. As such, the incorporation of orange oil increased antioxidant activity from 11.09% to 17.73%, cinnamon oil increased antioxidant activity from 9.36% to 15.98% and clove oil increased said activity from 8.36% to 17.27%. 

The antioxidant effects of various essential oils on films are not entirely known, given the scarcity of studies examining their effects on biopolymeric matrices. However, the differential antioxidant effects of various natural oils have been well documented. For example, the antioxidant properties of clove and cinnamon essential oils are comparable to those of BHT (butylhydroxytoluene), a chemical substance known for its protective effect against oxidation. These results were attributed to the high eugenol and β-caryophyllene content of these oils [34], with clove essential oil being one of the most powerful natural antioxidants, with effects even superior to those of BHT or butylated hydroxyanisole [35]. The high antioxidant activity and radical scavenging effect of eugenol is the result of its phenolic hydroxyl groups, which remain stable during the film development process [36]. Similarly, linalool, another chemical compound present in orange and cinnamon oils (Table S1), has been recognized for its antioxidant and antibacterial activity [37,38]. When examining the antioxidant activity of 25 essential oils used for medicinal purposes, Wei and Shibamoto [39] showed that clove (52%) and cinnamon (23%) oils had antioxidant activity superior to that of chamomile (20%), anise (20%), rosemary (10%) and orange (9%) EOs. Moreover, according to their study, mint essential oil, along with sandalwood and bergamot, had exhibited pro-oxidant activity. Therefore, our results of increased antioxidant activity caused by clove, cinnamon and orange essential oils are somewhat in line with the general antioxidant activity effects of these oils, albeit in a different model and likely due to the high eugenol and linalool contents (Table S1, Supplementary Files), as well as the high antioxidant capacity of EOs. Our data cannot explain the concentration differences in the antioxidant effects of these oils, which may be attributed, in part, to their varied embedding properties into the a biopolymeric matrix; however, this would require further investigation. 

As shown in Table 3, microorganisms proliferated (*TC*) from t0 to t1 (samples 1–7, 9–14 and 19). Except for samples 15–18, which did not show a microbiological load before or after storage, the other films showed colony-forming units on their surfaces. Only samples with 7.5% essential oil of lemon, clove and mint showed contamination with coliforms bacteria, whereas samples 4 (15% grapefruit oil) and 13 (7.5% chamomile oil) were contaminated with *Staphylococcus aureus*. No *Escherichia coli*, enterococcus, *Listeria monocytogenes*, yeasts or molds developed on the surfaces of the biopolymeric materials.

## 3. Conclusions

Films based on biopolymers have proven their applicability in various fields and can replace conventional materials based on non-renewable resources. The ease of incorporating various active substances into the matrix of films has contributed to their success. Essential oils present numerous benefits, both for the products packaged in such materials and in terms of the health of those who consume the edible films and coatings. Our results highlighted the use of essential oil favoring stability of the materials, even after one year of storage. The films with incorporated essential oils were preserved their properties after one year storage compared to the initial period. These positive effects were not present in the control samples. Therefore, the addition of plant-extracted oils to biopolymer products is associated with significant benefits, making them suitable for use in several industries. However, additional research is required to determine their mechanism of action, their prolonged effects and the synergistic effects that may occur when used in combination with other essential oils or biologically active substances, as well as their adverse effects.

## 4. Materials and Methods

The aim of this study was to develop, test and characterize new biopolymeric based films with incorporated EOs (Table 4). All substances used to produce 19 film-forming solutions (agar, sodium alginate, glycerol, emulsifier and Tween 80) were obtained from Sigma Aldrich (Germany). The commercially available EOs from medicinal plants, such as lemon, grapefruit, orange, cinnamon, clove, mint, eucalypt and chamomile, were obtained from Carl Roth (Germany). Ginger oil was purchased from Merk (Germany). According to the manufacturer data sheets, the essential oils were obtained by steam distillation; their chemical composition is summarized in Table S1.

Biopolymeric materials were obtained based on a previously developed and tested modified methodology [40]. Briefly, a ratio of 2:1:1 agar: alginate: glycerol was used. The film-forming solution was mixed for 20 min at 90 ± 2 °C and 450 rpm, cooled to 40 °C and 7.5 and 15% *w*/*v* essential oils were added. The solutions were obtained through the cast method after maintenance for 38–42 h at room temperature (26 ± 3 °C) and rH = 52 ± 3% until complete drying was achieved. 

The films were kept for one year in silicone paper packaging, protected from humidity and sunlight. They were tested immediately after development and after one year for physical and optical properties, as well as antioxidant and antimicrobial capacity. In order to observe possible variations in their mass, samples were weighed using an analytical balance. Film thickness (*t*, µm) was measured after five readings in different areas of the material surface using a Yato micrometer (Shanghai, China). The density (*D*, g/cm^3^) of the films was calculated by relating their mass (*w*) to the thickness (*t*) and surface (*a*) [41]:

(1) $$\mathrm{Density},\;\left(\frac{g}{{\mathrm{cm}}^{3}}\right)=\frac{w}{a*t}$$

Transmittance (*T*, %) and absorbance (*A*) were read spectrophotometrically (Epoch, BioTek Instruments, Winooski, VT, USA) in triplicate using 1 cm × 3 cm film samples. The transmittance was read in the wavelength range of 300 to 800 nm, with absorbance at 600 nm. An empty cuvette was used as a standard. The opacity of the material (*O*) was calculated according to the following formula:

(2) $$\mathrm{Opacity},\;\left(\frac{A}{\mathrm{mm}}\right)=A/t$$

where *A* = absorbance, and *t* = thickness (mm).

The water activity index (*a_w_*) was determined with AquaLab 4TE equipment (Meter Group, München, Germany) at 25 ± 0.7 °C. The results indicate the average of 5 readings of the tested materials. The evaluation of this parameter is of interest when film dehydration occurs. Additionally, a low value of the water activity index favors the prevention of the development and proliferation of microorganisms. A water activity index score above 0.7 is required to survive the environmental conditions.

The sample color was evaluated using a CIELab system with a Chroma Meter CR400 colorimeter (Konika Minolta, Tokyo, Japan). The results represent the arithmetic mean of ten readings taken over the entire surface of the material. In order to test the color difference between the samples tested before and after storage, the color deviation (*ΔE*) was calculated according to the following formula [42]:

(3) $$\Delta E=\sqrt{\left(\Delta L*\Delta L\right)+\left(\Delta a*\Delta a\right)+\left(\Delta b*\Delta b\right)}$$

The microstructure of images was visualized with a Celena microscope, and images and microtopographs were analyzed using Mountains Premium 9 (Digital Surf, Lavoisier, France).

The tensile strength (*TS*) and elongation (*E*) were tested with an ESM Mark 10 texturometer according to STAS ASTM D882 (Standard Test Method for Tensile Properties of Thin Plastic Sheeting) [43] and calculated according to Formulae (4) and (5) [44]. As such, a 5 KN cell and special grips for thin films and foils were attached, and 1cm × 10 cm film samples were tested. The travel speed was set at 10 mm/min, and the working temperature was 27.2 ± 0.2 °C.

(4) $$TS,\;\left(MPa\right)=\frac{F}{a}$$

where *F* is the maximum load (kN), and *a* is the surface (mm^2^). A travel speed of 10 mm/min was chosen based on standard requirements for testing films and foils of 5 to 10 mm/min, as well as on the published evidence [45,46,47,48,49,50].

The elongation at break (*E*) is the ratio between the final (*Δl*) and initial length (*l*) after test specimen breakage.

(5) $$E,\;\left(\%\right)=\frac{\Delta l}{l}*100$$

Antioxidants were assessed using a DPPH (2,2-diphenyl-1-picrylhydrazyl) radical scavenging assay according to the method described by Aloui et al. [51], with some modifications. Briefly, a film sample was cut into 20 mm × 20 mm, and 2 mL of DPPH was added, mixed for 1 min at 500 rpm and incubated at 35 °C for 30 min. After incubation, the absorbance was read at 517 nm using an Epoq spectrophotometer (BioTek Instruments, Winooski, VT, USA). The experiment was carried out in triplicate, and the radical scavenging activity was calculated according to Formula (6), where *Ac* is the absorbance of the DPPH solution without film, and *As* is absorbance of the sample:

(6) $$\mathrm{Radical}\;\mathrm{scaveging}\;\mathrm{activity},\;\left(\%\right)=\frac{Ac-As}{Ac}*100$$

The antimicrobial activity of the films was tested using specific plates (NISSUI Pharmaceutical, Tokyo, Japan) with dehydrated culture media. Thus, total count (*TC*), coliforms (*CF*), *Escherichia coli* (*EC*), *Staphylococcus aureus* (*X-SA*), *Listeria monocytogenes* (*LM*) and yeasts and molds (*YM*) were evaluated. The proposed method it is useful, as it faster than traditional methods and eliminates the risks that can intervene in the manipulation of strains of pathological microorganisms. It can also be used in laboratories in which the use of strains of pathogenic microorganisms is not allowed, as is our case. For the assay, 1 g of film was mixed with 9 mL saline solution at 500 rpm. Then, 1 mL of the solution was used to hydrate the culture medium. Later, plates with culture media were thermostated for 36–72 h at 37 °C. The results are expressed in CFU/g.

Statistical analyses were performed with one-way analysis of variance (ANOVA) and Tukey’s test. Significance was set at *p* < 0.05. Data analysis was performed using MiniTAB statistical software (MiniTAB Ltd., Coventry, UK). All determinations were made after sample development (*t*0) and after one year of storage (*t*1).

## Figures and Tables

**Table 1:** Optical parameters and water activity index at the time of development (t0) and after one year of storage (t1).

Sample	Density, g * cm^−1^		Opacity, A * mm^−1^		Water Activity Index, aw	
	t0	t1	t0	t1	t0	t1
1	0.71 ± 0.05 ^a^	0.66 ± 0.12 ^a^	3.31 ± 0.15 ^h^	4.01 ± 0.28 ^e^	0.786 ± 0.05 ^a^	0.334 ± 0.15 ^c^
2	0.76 ± 0.06 ^a^	0.65 ± 0.17 ^a^	3.82 ± 0.01 ^e^	3.61 ± 0.25 ^j^	0.743 ± 0.05 ^b^	0.286 ± 0.59 ^d^
3	0.55 ± 0.10 ^e,f^	0.39 ± 0.16 ^d,e,f^	7.77 ± 0.01 ^a^	3.83 ± 0.42 ^g,h^	0.714 ± 0.01 ^d^	0.304 ± 0.15 ^c,d^
4	0.74 ± 0.02 ^a^	0.42 ± 0.11 ^c,d^	4.85 ± 0.09 ^c^	3.86 ± 0.18 ^f,g^	0.737 ± 0.05 ^c^	0.298 ± 0.15 ^c,d^
5	0.54 ± 0.12 ^e,f^	0.43 ± 0.12 ^c,d^	2.07 ± 0.12 ^j^	3.62 ± 0.02 ^i,j^	0.738 ± 0.07 ^c^	0.302 ± 0.10 ^c,d^
6	0.56 ± 0.17 ^d,e,f^	0.46 ± 0.54 ^c^	3.66 ± 0.17 ^f,g^	3.97 ± 0.14 ^e,f^	0.700 ± 0.03 ^e^	0.295 ± 0.03 ^c,d^
7	0.58 ± 0.10 ^c,d,e^	0.42 ± 0.12 ^c,d^	1.47 ± 0.02 ^l^	4.21 ± 0.06 ^d^	0.702 ± 0.05 ^e^	0.332 ± 0.10 ^c^
8	0.64 ± 0.19 ^b,c^	0.38 ± 0.81 ^d,e,f^	1.84 ± 0.01 ^k^	4.32 ± 0.02 ^d^	0.685 ± 0.07 ^g^	0.304 ± 0.26 ^c,d^
9	0.57 ± 0.16 ^d,e,f^	0.43 ± 0.21 ^c,d^	1.73 ± 0.04 ^k^	3.73 ± 0.02 ^h,i^	0.574 ± 0.02 ^k^	0.292 ± 0.15 ^c,d^
10	0.42 ± 0.13 ^g^	0.25 ± 0.38 ^g^	1.85 ± 0.01 ^k^	5.71 ± 0.10 ^b^	0.571 ± 0.06 ^k^	0.272 ± 0.05 ^d^
11	0.62 ± 0.10 ^b,c,d^	0.43 ± 0.15 ^c,d^	7.03 ± 0.01 ^b^	2.03 ± 0.02 ^l,m^	0.587 ± 0.75 ^j^	0.517 ± 0.02 ^b^
12	0.65 ± 0.15 ^b^	0.40 ± 0.17 ^d,e^	3.57 ± 0.03 ^g^	1.97 ± 0.08 ^m,n^	0.751 ± 0.01 ^b^	0.524 ± 0.02 ^a,b^
13	0.51 ± 0.17 ^f^	0.34 ± 0.15 ^f^	3.74 ± 0.03 ^e,f^	6.47 ± 0.02 ^a^	0.706 ± 0.75 ^e^	0.564 ± 0.04 ^a^
14	0.53 ± 0.15 ^f^	0.42 ± 0.10 ^c,d^	1.83 ± 0.03 ^k^	5.27 ± 0.03 ^c^	0.684 ± 0.69 ^g^	0.562 ± 0.02 ^a^
15	0.64 ± 0.30 ^b,c^	0.54 ± 0.21 ^b^	1.55 ± 0.07 ^l^	1.83 ± 0.03 ^o^	0.656 ± 0.01 ^h^	0.561 ± 0.02 ^a^
16	0.76 ± 0.42 ^a^	0.66 ± 0.26 ^a^	0.95 ±0.01 ^m^	1.14 ± 0.02 ^p^	0.692 ± 0.05 ^f^	0.546 ± 0.05 ^a,b^
17	0.43 ± 0.02 ^g^	0.35 ± 0.02 ^e,f^	1.53 ± 0.31 ^l^	2.12 ± 0.05 ^l^	0.655 ± 0.75 ^h^	0.534 ± 0.03 ^a,b^
18	0.52 ± 0.15 ^e,f^	0.42 ± 0.13 ^c,d^	2.54 ± 0.03 ^i^	1.92 ± 0.10 ^n,o^	0.645 ± 0.05 ^i^	0.527 ± 0.03 ^a,b^
19	0.44 ± 0.35 ^g^	0.34 ± 0.21 ^f^	4.41 ± 0.11 ^d^	2.51 ± 0.10 ^k^	0.646 ± 0.01 ^i^	0.281 ± 0.01 ^d^

* The values represent the mean ± SD. Means that do not share the same superscript (a–p) are significantly different (*p* < 0.05).

**Table 2:** Evaluation of color parameters before (t0) and after storage (t1).

Sample	L*		a*		b*		ΔE
	t0	t1	t0	t1	t0	t1	
1	92.73 ± 0.13 ^a,b^	88.79 ± 0.18 ^b,c^	−5.51 ± 0.04 ^a^	−0.49 ± 0.02 ^a^	11.42 ± 0.17 ^h^	10.76 ± 0.31 ^f^	6.42 ± 0.01 ^f^
2	92.23 ± 0.5 ^a,b,c^	88.99 ± 0.16 ^b,c^	−5.82 ± 0.08 ^f,g^	−0.42 ± 0.24 ^a^	13.43 ± 0.67 ^a,b,c^	10.61 ± 0.30 ^f^	6.53 ± 0.04 ^f^
3	92.12 ± 0.16 ^a,b,c^	88.30 ± 0.40 ^b,c,d^	−5.90 ± 0.02 ^g,h^	−0.54 ± 0.02 ^a^	12.93 ± 0.33 ^c,d^	11.33 ± 0.48 ^d,e,f^	6.92 ± 0.01 ^d^
4	92.42 ± 0.28 ^a,b,c^	88.87 ± 0.96 ^b,c^	−5.97 ± 0.08 ^h^	−0.61 ± 0.05 ^a^	14.08 ± 0.5 ^a^	10.72 ± 0.53 ^f^	7.55 ± 0.02 ^b^
5	92.45 ± 0.28 ^a,b,c^	88.88 ± 0.73 ^b,c^	−5.78 ± 0.04 ^e,f^	−0.49 ± 0.04 ^a^	12.5 ± 0.19 ^d. e. f^	8.85 ± 0.20 ^g^	7.25 ± 0.05 ^c^
6	92.92 ± 0.31 ^a^	87.17 ± 0.63 ^b^	−5.73 ± 0.02 ^d,e,f^	−0.48 ± 0.15 ^a^	12.82 ± 0.08 ^c,d,e^	9.85 ± 0.73 ^f,g^	6.97 ± 0.02 ^d^
7	92.47± 0.32 ^a,b,c^	89.17 ± 0.30 ^b^	−5.68 ± 0.04 ^c,d,e^	−0.48 ± 0.01 ^a^	11.36 ± 0.30 ^h^	10.18 ± 0.08 ^d,e,f^	6.51 ± 0.03 ^f^
8	92.57 ± 0.16 ^a,b,c^	88.58 ± 0.92 ^b,c^	−5.66 ± 0.01 ^b,c,d^	−0.49 ± 0.02 ^a^	11.84 ± 0.30 ^f,g,h^	10.95 ± 0.42 ^f^	6.52 ± 0.03 ^f^
9	92.41 ± 0.39 ^a,b,c^	88.50 ± 0.94 ^b,c,d^	−5.60 ± 0.01 ^b,c,d^	−0.47 ± 0.03 ^a^	12.50 ± 0.27 ^d,e,f^	11.14 ± 1.00 ^e,f^	6.74 ± 0.02 ^e^
10	92.46 ± 0.21 ^a,b,c^	88.31 ± 0.68 ^b,c,d^	−5.64 ± 0.03 ^b,c,d^	−0.52 ± 0.01 ^a^	11.34 ± 0.31 ^h^	11.22 ± 0.40 ^d,e,f^	6.24 ± 0.06 ^g^
11	90.61 ± 0.64 ^d^	92.25 ± 0.80 ^a^	−5.52 ± 0.03 ^a^	−5.78 ± 0.07 ^c^	13.30 ± 0.04 ^b,c^	13.70^,^± 0.53 ^b,c^	1.25 ± 0.01 ^k^
12	91.90 ± 0.48 ^b,c^	92.18 ± 0.15 ^a^	−5.60 ± 0.03 ^a,b,c^	−5.79 ± 0.02 ^c^	12.50 ± 0.13 ^d,e,f^	12.84 ± 0.52 ^c,d^	0.24 ± 0.15 ^m^
13	92.65 ± 0.24 ^a,b^	91.00 ± 0.64 ^a^	−5.59 ± 0.02 ^a,b,c^	−5.76 ± 0.01 ^c^	12.51 ± 0.05 ^d,e,f^	15.01 ± 0.40 ^c^	3.63 ± 0.02 ^h^
14	92.34 ± 0.11 ^a,b,c^	92.08 ± 0.28 ^a^	−5.67 ± 0.03 ^b,c,d^	−5.85 ± 0.03 ^c^	12.18 ± 0.01 ^e,f,g^	13.61 ± 0.37 ^b,c^	0.85 ± 0.01 ^l^
15	92.54 ± 0.14 ^a,b,c^	92.27 ± 0.24 ^a^	−5.68 ± 0.06 ^c,d,e^	−5.85 ± 0.03 ^c^	12.50 ± 0.38 ^d,e,f^	13.17 ± 0.40 ^c^	0.83 ± 0.02 ^l^
16	91.80 ± 0.40 ^c^	91.83 ± 0.15 ^a^	−5.81 ± 0.01 ^f,g^	−5.76 ± 0.08 ^c^	13.82 ± 0.02 ^a,b^	15.06 ± 0.27 ^b^	1.61 ± 0.02 ^j^
17	92.41 ± 0.19 ^a,b,c^	92.09 ± 0.44 ^a^	−5.68 ± 0.03 ^c,d,e^	−5.91 ± 0.10 ^c^	11.53 ± 0.15 ^g,h^	12.84 ± 0.67 ^c,d,e^	2.25 ± 0.04 ^i^
18	92.42 ± 0.33 ^a,b,c^	86.97 ± 1.09 ^d^	−5.58 ± 0.04 ^a,b^	−5.18 ± 0.23 ^b^	11.75 ± 0.18 ^g,h^	21.97 ± 0.8 ^a^	11.35 ± 0.21 ^a^
19	92.51 ± 0.12 ^a,b,c^	87.47 ± 0.22 ^c,d^	−5.64 ± 0.02 ^b,c,d^	−0.62 ± 0.02 ^a^	11.32 ± 0.09 ^h^	10.32 ± 0.56 ^f,g^	7.14 ± 0.02 ^c^

L*, lightness; a*, green-to-red parameter; b*, blue-to-yellow parameter. The values represent mean ± SD. Means that do not share the same superscript (a–m) are significantly different (*p* < 0.05).

**Table 3:** Microbiological assessments before (t0) and after storage (t1).

Sample	*TC*		*EC*		*ETC*		*CF*		*YM*		*X-SA*		*LM*	
	t0	t1	t0	t1	t0	t1	t0	t1	t0	t1	t0	t1	t0	t1
1	1	15	-	-	-	-	1	13	-	-	-	-	-	-
2	-	7	-	-	-	-	-	-	-	-	-	-	-	-
3	15	22	-	-	-	-	-	-	-	-	-	-	-	-
4	21	42	-	-	-	-	-	-	-	-	1	6	-	-
5	-	6	-	-	-	-	-	-	-	-	-	-	-	-
6	7	31	-	-	-	-	-	-	-	-	-	-	-	-
7	2	4	-	-	-	-	-	-	-	-	-	-	-	-
8	-	-	-	-	-	-	-	-	-	-	-	-	-	-
9	13	48	-	-	-	-	2	7	-	-	-	-	-	-
10	1	13	-	-	-	-	-	-	-	-	-	-	-	-
11	-	9	-	-	-	-	2	5	-	-	-	-	-	-
12	8	11	-	-	-	-	-	-	-	-	-	-	-	-
13	19	64	-	-	-	-	-	-	-	-	1	4	-	-
14	6	34	-	-	-	-	-	-	-	-	-	-	-	-
15	-	-	-	-	-	-	-	-	-	-	-	-	-	-
16	-	-	-	-	-	-	-	-	-	-	-	-	-	-
17	-	-	-	-	-	-	-	-	-	-	-	-	-	-
18	-	-	-	-	-	-	-	-	-	-	-	-	-	-
19	28	114	-	-	-	-	-	-	-	-	-	-	-	-

TC, total count; EC, Escherichia coli; ETC, Enterococcus; CF, coliform; YM, yeasts and molds; X-SA, Staphylococcus aureus; LM, Listeria monocytogenes. All results are expressed in CFU/g.

**Table 4:** Composition of new biopolymer-based films.

Sample	7.5% EO (*w*/*v*)	15% EO (*w*/*v*)	Sample	7.5% EO (*w*/*v*)	15% EO (*w*/*v*)
1	lemon	x	11	mint	x
2	x	lemon	12	x	mint
3	grapefruit	x	13	chamomile	x
4	x	grapefruit	14	x	chamomile
5	orange	x	15	ginger	x
6	x	orange	16	x	ginger
7	cinnamon	x	17	eucalyptus	x
8	x	cinnamon	18	x	eucalyptus
9	clove	x	19	Control, no essential oil added	
10	X	clove			

## Data Availability

Not applicable.

## Supplementary Materials

The following supporting information can be downloaded at: https://www.mdpi.com/article/10.3390/gels8110756/s1, Figure S1: Microstructure images of tested films, obtained at initial time (*t*0) and after one year storage (*t*1); Table S1. Essential oils’ chemical composition, according to the manufacturer data sheet
