# Supplementary material for: The Use of Biopolymers as a Natural Matrix for Incorporation of Essential Oils of Medicinal Plants
Source: Gels. 2022 Nov 21;8(11):756. doi: 10.3390/gels8110756 (PMC9690358; doi:10.3390/gels8110756)
Supplement: Supplementary file 1 [file gels-08-00756-s001.zip › gels-2010860-supplementary.pdf]

Microscope image *t0*	Microscope image *t1*	Microtopography *t0*	Microtopography *t1*
**Sample 1 (7.5% lemon EO)**			
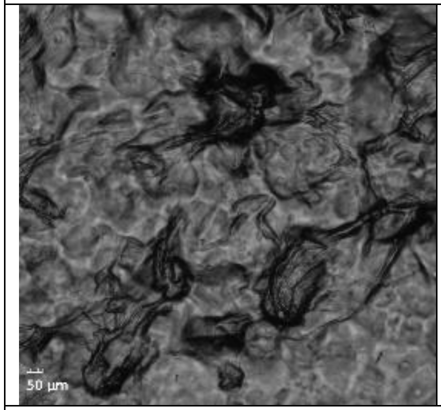	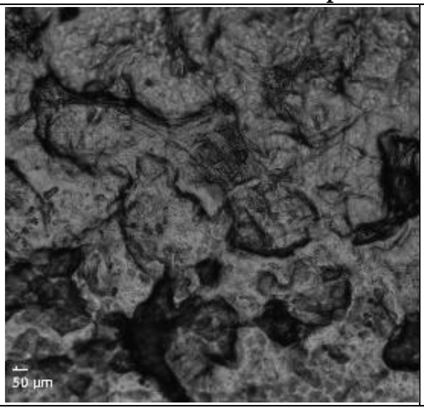	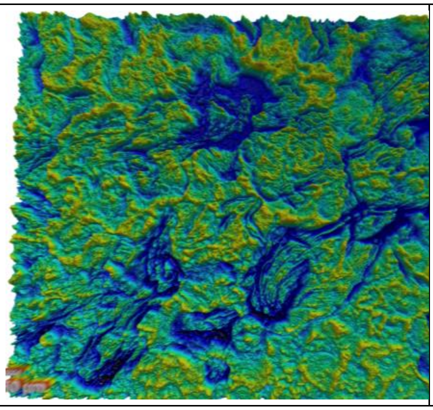	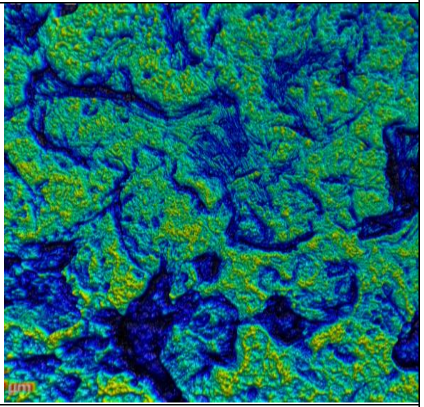
**Sample 2 (15% lemon EO)**			
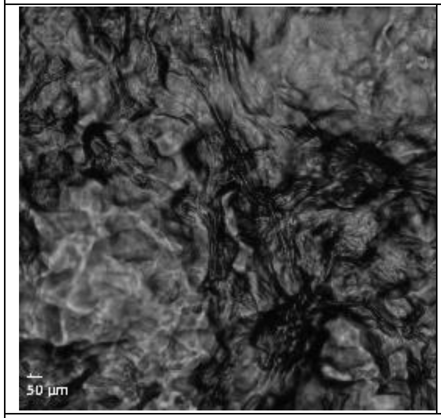	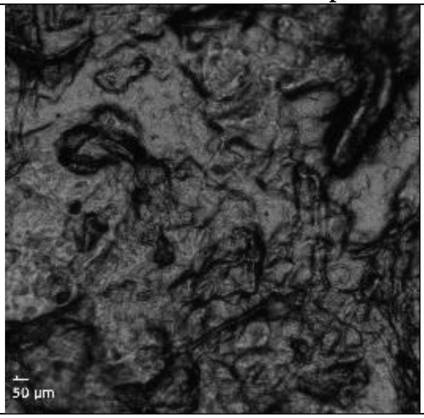	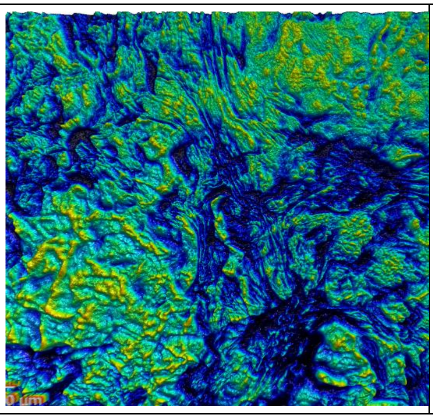	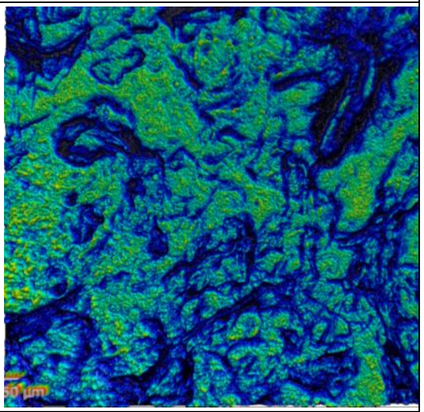
**Sample 3 (7.5% grapefruit EO)**			

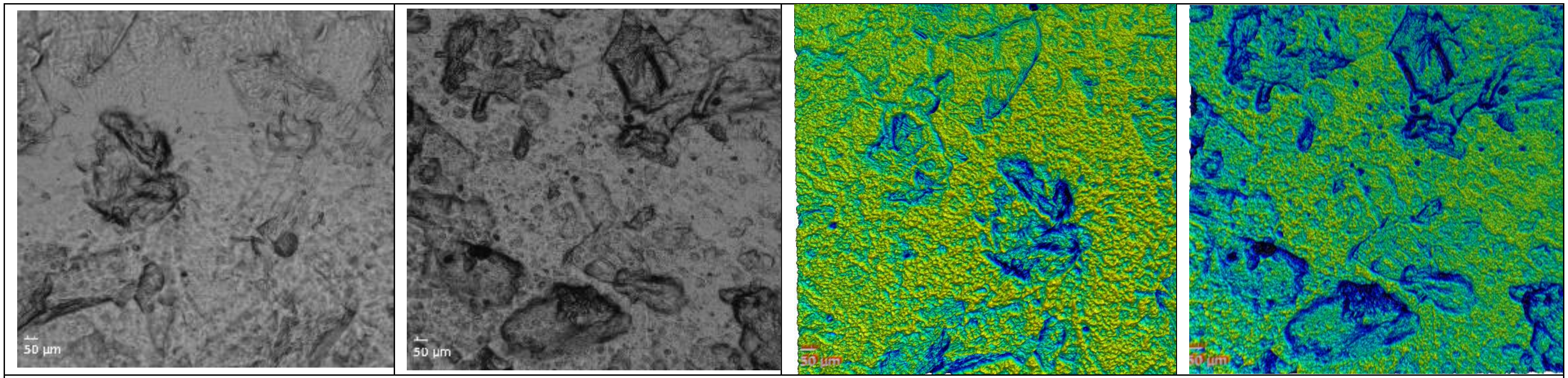

Sample 4 (15% grapefruit EO)

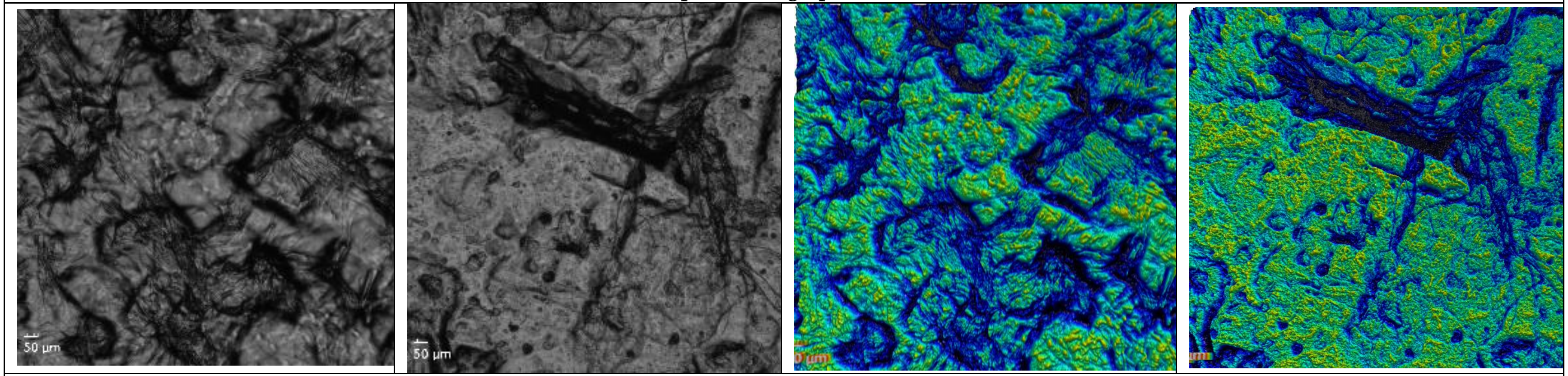

Sample 5 (7.5% orange EO)

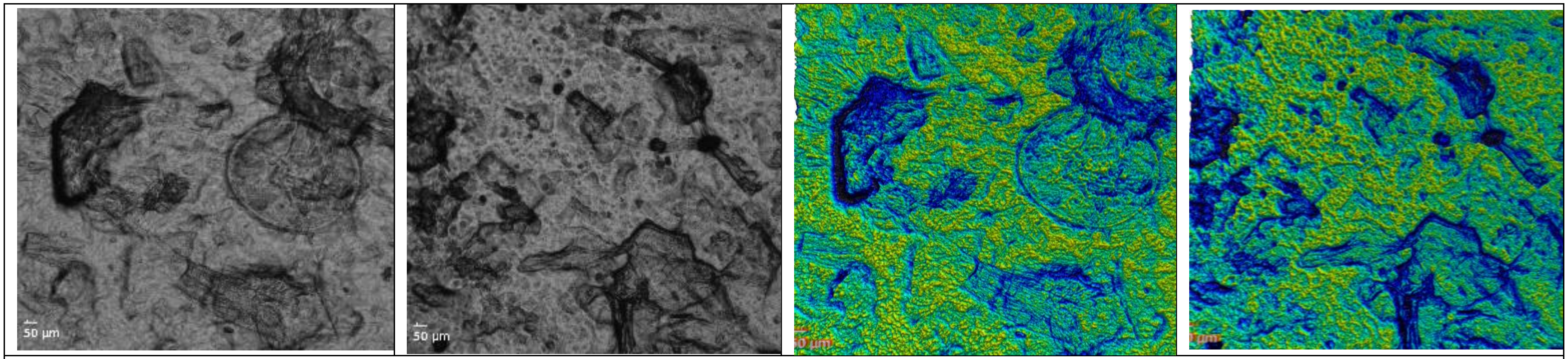

Sample 6 (15% orange EO)

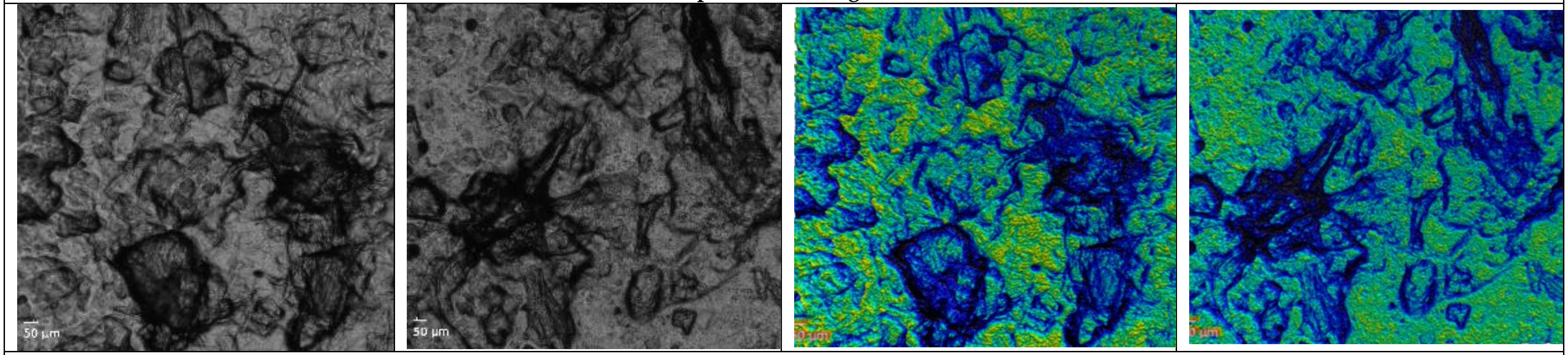

Sample 7 (7.5% cinnamon EO)

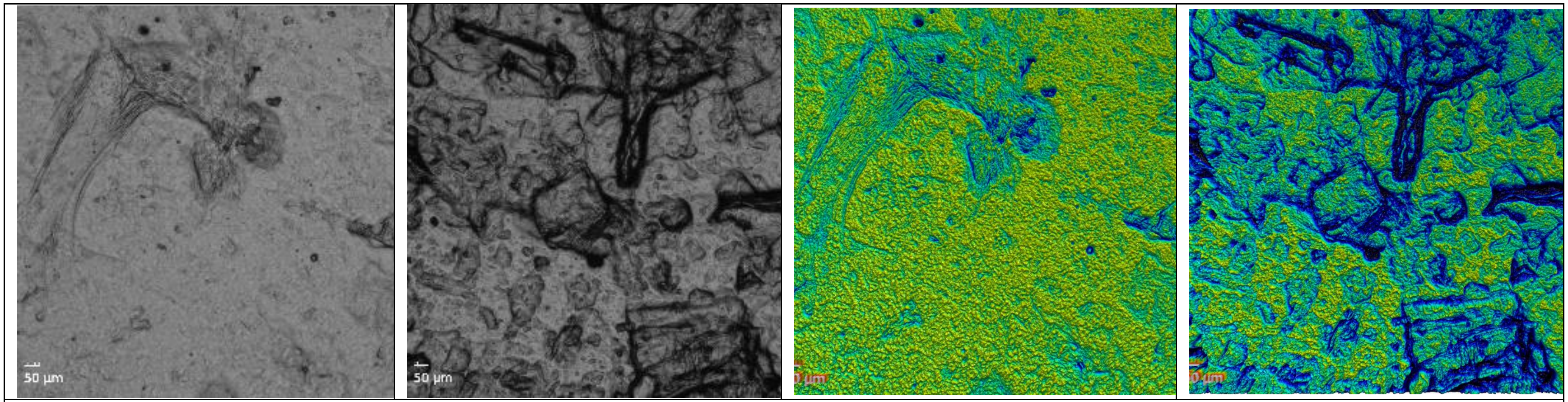

Sample 8 (15% cinnamon EO)

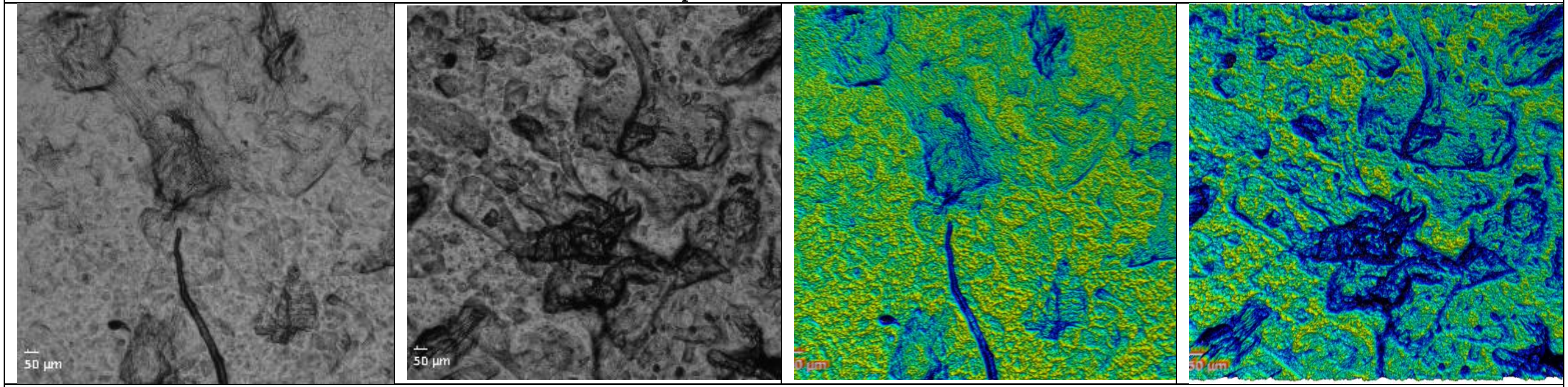

Sample 9 (7.5% clove EO)

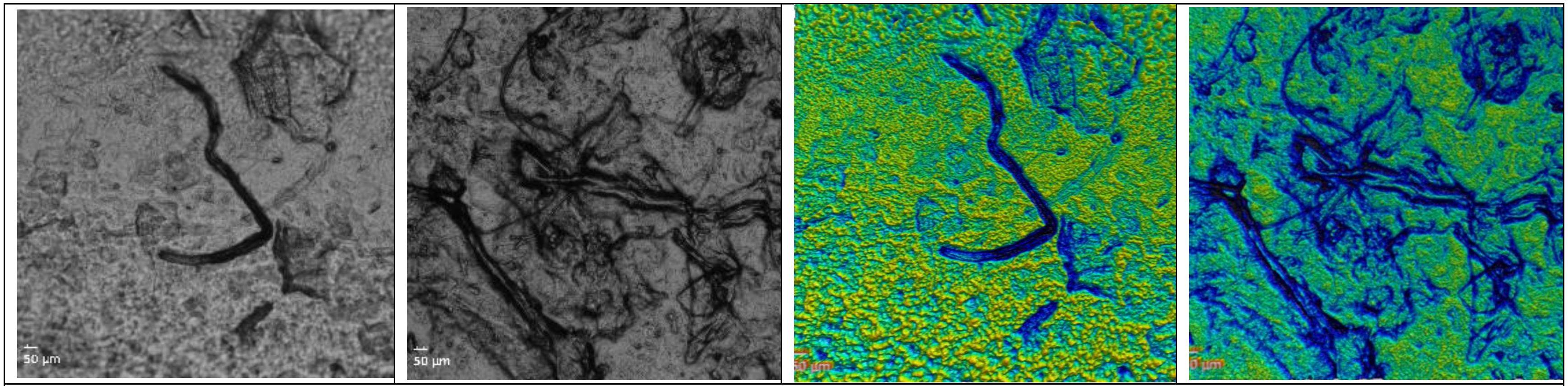

Sample 10 (15% clove EO)

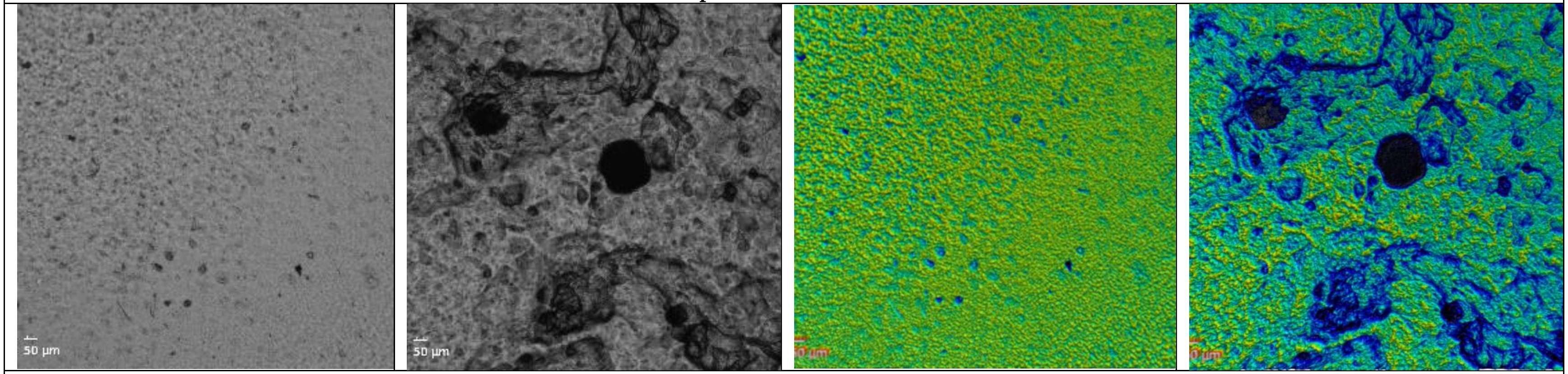

Sample 11 (7.5% mint EO)

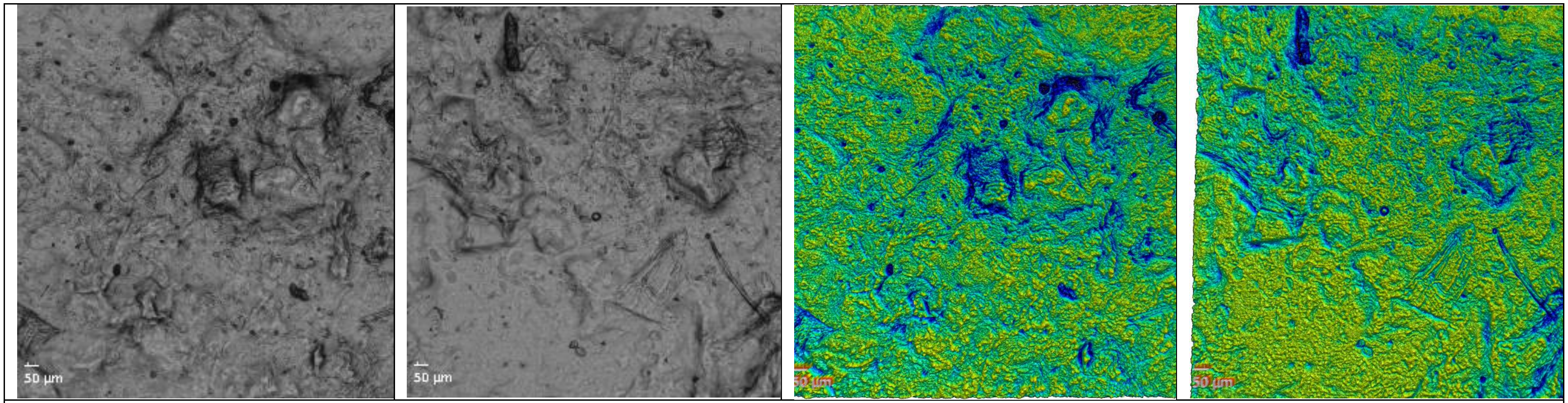

Sample 12 (15% mint EO)

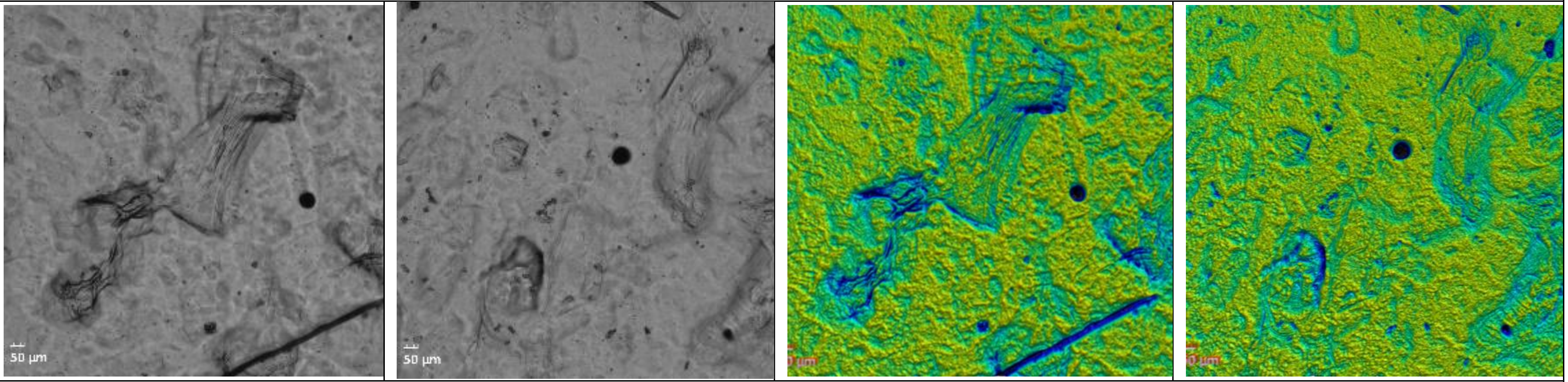

Sample 13 (7.5% ginger EO)

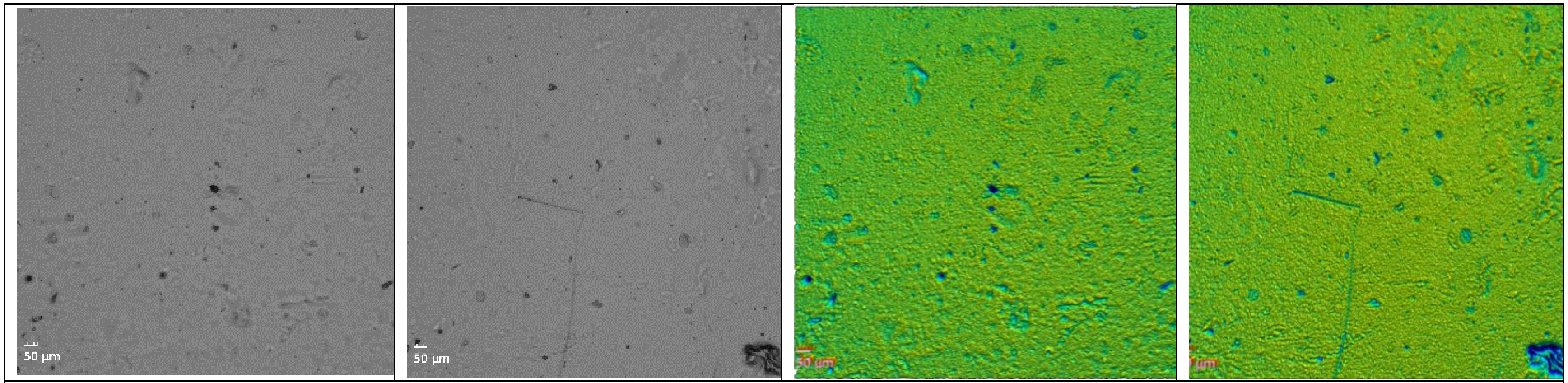

Sample 14 (15% ginger EO)

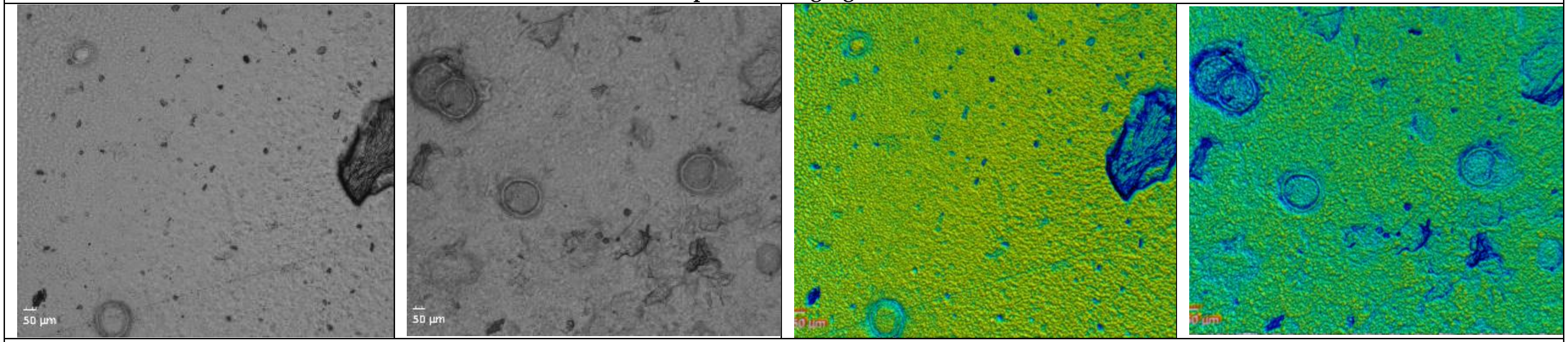

Sample 15 (7.5% eucalyptus EO)

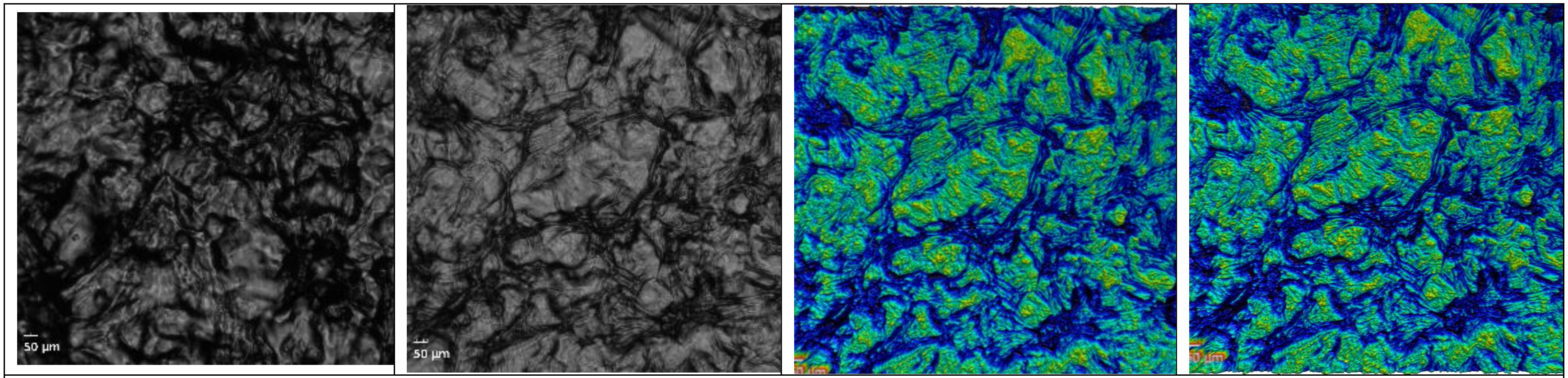

Sample 16 (15% eucalyptus EO)

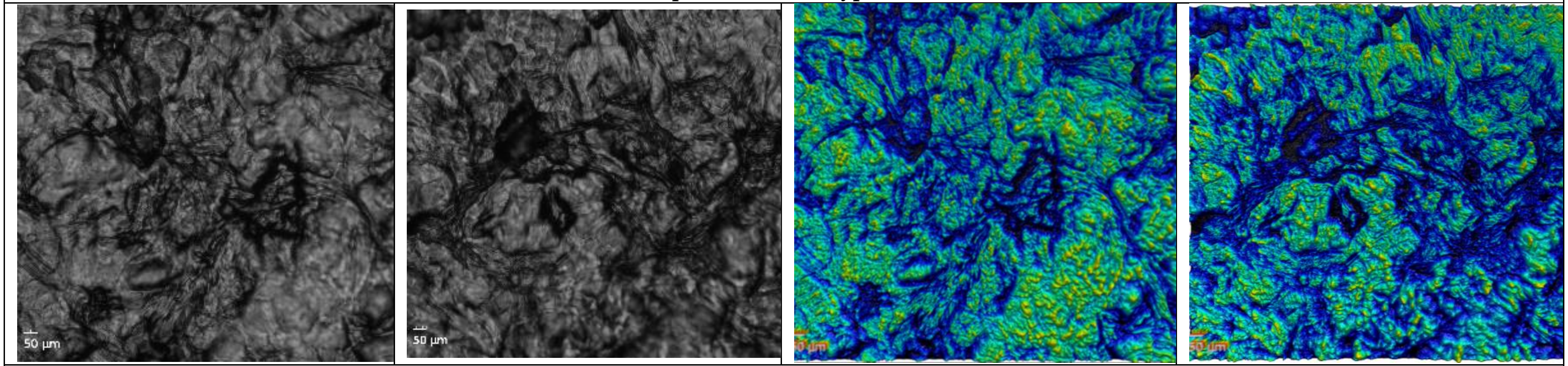

Sample 17 (7.5% chamomile EO)

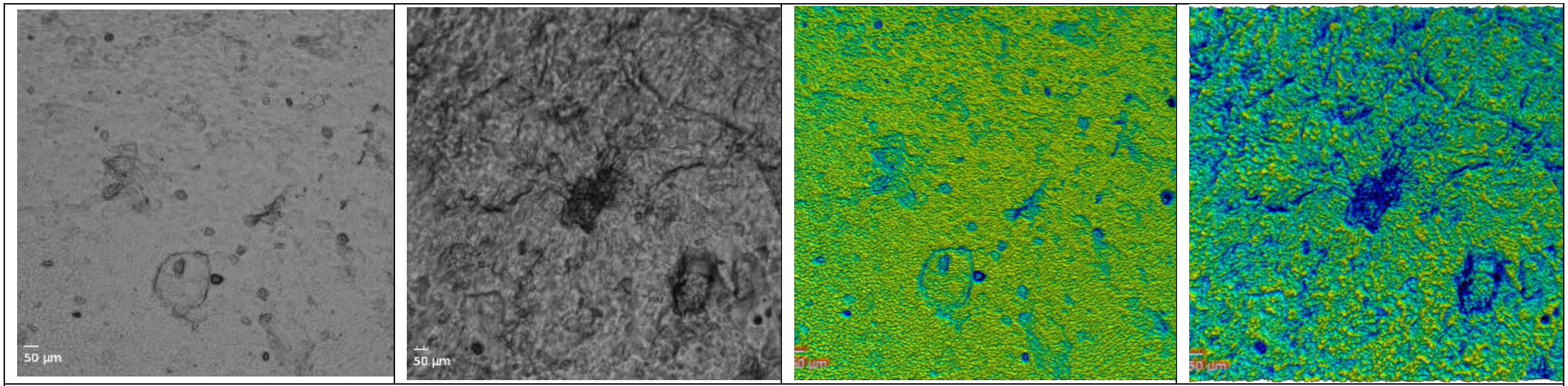

Sample 18 (15% chamomile EO)

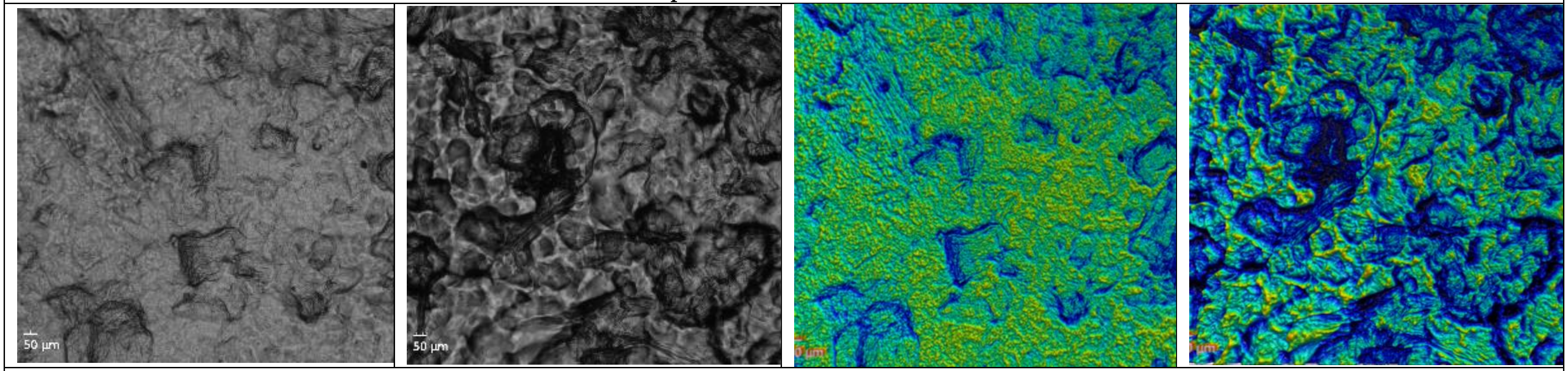

Sample 19 (control, no EO)

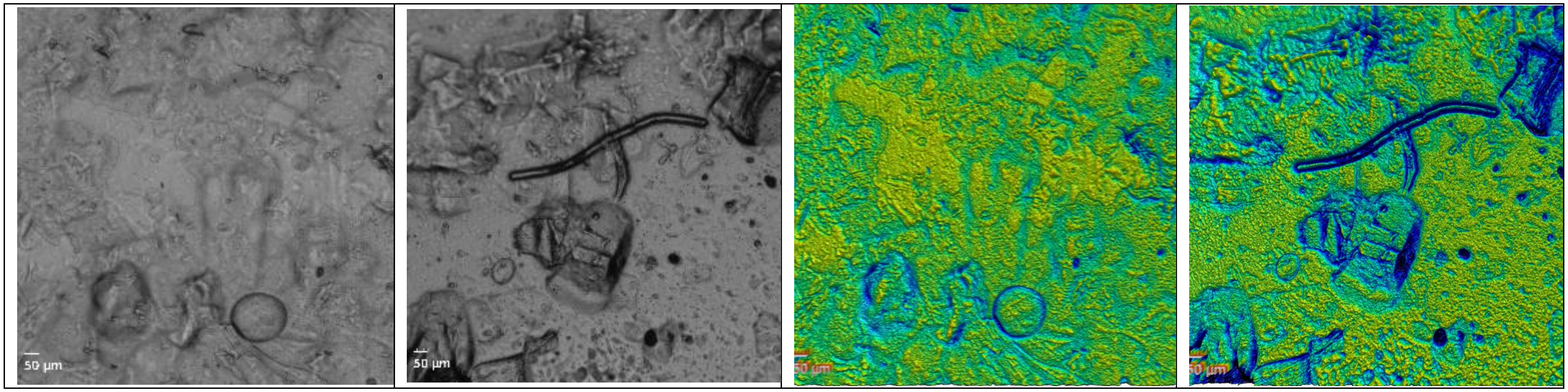

Figure S1. Microstructure images of tested films, obtained at initial time (*t0*) and after one year storage (*t1*).

Table S1. Essential oils' chemical composition, according to the manufacturer data sheet.

Essential oil	*Chemical composition*
lemon	ß-Pinene, γ-Terpinene, D-(+)-Limonene, Citral, Myrcene
orange	DL-α-Pinene, Linalool, Citral, Myrcene,
grapefruit	D-(+)-Limonene, Myrcene
clove	Eugenol, β-Caryophyllene, α-Humulene, Isoeugenol
cinnamon	Eugenol, Linalool, DL-α-Pinene, DL-Limonene
chamomile	Camphene
mint	(-)-Menthol, (-)-Menthone, Eucalyptol, L-limonene, ß-Pinene, DL-α-Pinene
eucalyptus	Eucalyptol, D-(+)-Limonene, DL-α-Pinene, (+)- Camphor, Myrcene, ß-Pinene, Sabinene, α-Terpineol, γ-Terpinene
ginger	Geranial, Neral, Camphene, Zingiberene, ar-curcumene, β-bisabolene, β-sesquiphellandrene
